# Iberdomide, ixazomib and dexamethasone in elderly patients with multiple myeloma at first relapse

**DOI:** 10.1111/bjh.19978

**Published:** 2025-01-05

**Authors:** Cyrille Touzeau, Xavier Leleu, Mourad Tiab, Margaret Macro, Aurore Perrot, Julie Gay, Carine Chateleix, Stéphane Moreau, Lionel Karlin, Caroline Jacquet, Salomon Manier, Cyrille Hulin, Olivier Decaux, Valentine Richez, Thomas Chalopin, Mohamad Mohty, Frédérique Orsini-Piocelle, Denis Caillot, Cécile Sonntag, Marguerite Vignon, Arthur Bobin, Hervé Avet-Loiseau, Alexandra Jobert, Lucie Planche, Jill Corre, Philippe Moreau

**Affiliations:** 1 Service d’hématologie, Centre Hospitalo-Universitaire, Nantes, France; 2 CRCINA, INSERM, CNRS, Université d’Angers, Université de Nantes, Nantes, France; 3 Site de Recherche Intégrée sur le Cancer (SIRIC) « ILIAD », INCA-DGOS-Inserm_12558, Nantes, France; 4 Service d’hématologie, Centre Hospitalo-Universitaire, Université de Poitiers, Poitiers, France; 5 Service d’hématologie, Centre Hospitalier Departmental, La Roche sur Yon, France; 6 Service d’hématologie, Centre Hospitalo-Universitaire, Caen, France; 7 Service d’hématologie, Centre Hospitalo-Universitaire, Institut Universitaire du Cancer Toulouse Oncopole, Université Paul Sabatier, Toulouse, France; 8 Service d’hématologie, Centre Hospitalier, Bayonne, France; 9 Service d’hématologie, Centre Hospitalo-Universitaire, Clermont-Ferrand, France; 10 Service d’hématologie, Centre Hospitalo-Universitaire, Limoges, France; 11 Service d’hématologie, Hôpital Lyon Sud, Pierre-Benite, France; 12 Service d’hématologie, Centre Hospitalo-Universitaire de Nancy, Vandoeuvre-lès-Nancy, France; 13 Maladies du Sang, Centre Hospitalo-Universitaire, Lille, France; 14 service d’hématologie, Hôpital Haut-Lévêque, Centre Hospitalo-Universitaire de Bordeaux, Pessac, France; 15 Service d’hématologie, Centre Hospitalo-Universitaire, Rennes, France; 16 Service d’hématologie, Centre Hospitalo-Universitaire, Nice, France; 17 Service d’hématologie, Centre Hospitalo-Universitaire, Tours, France; 18 Service d’hématologie, Hôpital Saint Antoine, Paris, France; 19 Service d’hématologie, Centre Hospitalier Regional, Annecy, France; 20 Hématologie Clinique, Centre Hospitalo-Universitaire, Dijon, France; 21 Hématologie Clinique, Institut de Cancérologie de Strasbourg Europe, Strasbourg, France; 22 Service d’hématologie, Hôpital Cochin, Paris, France; 23 Département de Recherche Clinique, Centre Hospitalo-Universitaire, Nantes, France

**Keywords:** elderly, iberdomide, ixazomib, myeloma

## Abstract

Most transplant-ineligible patients present with multiple myeloma (MM) refractory to lenalidomide and/or anti-CD38 monoclonal antibody at first relapse and represent a difficult-to-treat population. The Intergroupe Francophone du Myélome phase 2 study iberdomide, ixazomib and dexamethasone (I2D) evaluated the oral triplet iberdomide, ixazomib and dexamethasone in MM patients aged ≥70 years at first relapse (NCT04998786). Seventy patients were enrolled to receive iberdomide (1.6 mg on day 1–21), ixazomib (3 mg on day 1, 8, 15) and dexamethasone (20 mg on day 1, 8, 15, 22 on cycle 1–2 and 10 mg on day 1, 8, 15, 22 on cycle 3–6) (28-day cycle) until disease progression. Median age was 76; 50% patients were frail according to the International Myeloma Working Group frailty score; 74% and 37% were refractory to lenalidomide and daratumumab respectively. With a median follow-up of 14 months, the overall response rate was 64%, including 36% very good partial response or better. The 12-month progression-free survival, duration of response and overall survival were 52%, 76% and 86% respectively. The most common (46%) grade 3–4 toxicity was neutropenia. Non-haematological adverse events were mostly grade 1 or 2. Overall, I2D demonstrated a favourable risk–benefit profile in elderly MM patients at first relapse, including in patients with lenalidomide and daratumumab refractory disease.

## INTRODUCTION

The triplet combinations bortezomib, lenalidomide, dexamethasone (VRd) and daratumumab, lenalidomide, dexamethasone (DRd) given until disease progression are standard of care for the treatment of transplant ineligible (TI) patients with newly diagnosed multiple myeloma (NDMM).^1–3^ Therefore, most TI MM patients present with disease refractory to lenalidomide and/or anti-CD38 at first relapse and represent a difficult-to-treat population. In lenalidomide refractory patients, pomalidomide, bortezomib and dexamethasone (PVd) represents an effective anti-CD38-free combination in relapsed/refractory multiple myeloma (RRMM) patients.^4^ However, bortezomib is associated with a risk of peripheral neuropathy and the resulting morbidities, especially in elderly patients. Iberdomide is an oral novel cereblon E3 ligase modulator (CELMoD) that binds cereblon with higher affinity, inducing the closed conformation required for more rapid degradation and greater potency compared with immunomodulatory drugs.^5,6^ Iberdomide demonstrated promising efficacy with a favourable safety profile in multi-class (including lenalidomide and pomalidomide) refractory patients.^7^ Ixazomib is an oral proteasome inhibitor approved for the treatment of RRMM associated with a reduced risk of peripheral neuropathy.^8^ Based notably on the results of phase 2 trials, ixazomib is recommended in combination with pomalidomide for lenalidomide-refractory patients at first relapse.^9–11^ We report here the results of a prospective, open-label, multicentre, phase 2 study evaluating the feasibility and efficacy of iberdomide, ixazomib and dexamethasone (I2D) in older patients at first relapse.

## METHODS

### Patients

Adult patients aged 70 years or older with MM at first relapse were eligible for enrolment. Patients had RRMM according to International Myeloma Working Group (IMWG) criteria.^12^ Patients had received one prior line of therapy (defined as ≥3 cycles of each therapy) and had Eastern Cooperative Oncology Group (ECOG) performance status ≤2. Other key inclusion criteria were adequate renal function (creatinine clearance ≥30 mL/min), absolute neutrophil count ≥1000/mm^3^ and platelets ≥75 000/mm^3^. Key exclusion criteria were disease refractory to bortezomib, prior treatment with pomalidomide or iberdomide. Full eligibility criteria are indicated in the study protocol (Supplementary data). All patients provided written informed consent.

### Study design

This prospective, multicentre, single-arm, open-label, phase 2 study was conducted at 22 centres of the Intergroupe Francophone du Myélome (IFM). The study was approved by the relevant national health authority agency and the National Ethics Advisory Committee and was conducted in accordance with the International Conference on Harmonization of Good Clinical Practice guidelines and the principles of the Declaration of Helsinki. This clinical trial is registered at www.clinicaltrials.gov as NCT04998786. All patients received oral iberdomide (1.6mg on day 1–21) and oral ixazomib (3 mg on day 1, 8, 15) until disease progression or unacceptable toxicity. Treatment was administered in 28-day cycles. The starting dose of iberdomide was the recommended phase 2 dose determined by the CC-220-MM-001 study.^7^ The starting dose of ixazomib (3 mg instead of 4 mg) was chosen to limit the risk of thrombocytopenia. In order to optimize safety in this elderly population, oral dexamethasone was given during a short duration (six cycles) with rapid dose reduction (20mg on day 1, 8, 15, 22 on cycle 1–2 and 10 mg on day 1, 8, 15, 22 on cycle 3–6). All patients received anticoagulant treatment for prevention of deep vein thrombosis (DVT) with low molecular weight heparin or low-dose aspirin, anti-viral therapy for herpes zoster prevention and antibiotic prophylaxis for bacterial infections.

### Study objectives and assessments

The primary objective was the rate of patients achieving at least very good partial response (VGPR). Secondary endpoints included overall response, duration of response (DOR), progression-free survival (PFS), overall survival (OS) and safety. Myeloma response assessment was based on the modified IMWG uniform response criteria.^13^ Complete response (CR) was defined as negative serum and urine immunofixation, absence of soft tissue plasmacytoma, normal calcium concentration and less than 5% plasma cells in the bone marrow. Stringent CR (sCR) was defined with the above criteria plus a normal serum free light chain (FLC) ratio. All patients were followed until death or end of the study. Frailty was determined using the IMWG frailty score.^14^ Safety was monitored until 30 days after the last dose of study drug, except for secondary malignancies which were monitored continuously during follow-up. Toxicities were graded according to the National Cancer Institute Common Toxicity Criteria of Adverse Events (version 5.0; Bethesda, MD).

### Statistical analysis

The goal of this programme was to validate the efficacy of I2D in older patients with MM at first relapse. We considered this therapy as sufficiently efficient provided that at least 45% of patients were able to achieve a VGPR or better. A sample size of 70 patients was required to estimate an efficacy rate of 55% to within a 95% confidence interval (CI) of ±10% (95% CI 45%-65%). Median follow-up duration was estimated using the reverse Kaplan-Meier method. PFS was calculated as the time from start of treatment to the first documentation of progressive disease, or death if the patient died due to any cause before progression. DOR was calculated as the time from achievement of at least partial response to the first documentation of progressive disease, or death if the patient died due to any cause before progression. OS was calculated as the time from the start of treatment to death. The Kaplan–Meier method was used to estimate the survival distribution. All analyses were conducted using R-version 4.2.3.

## RESULTS

### Patients

Between December 2021 and May 2023, 70 eligible patients with MM at first relapse were enrolled at 20 centres in France. Patients’ demographic and baseline disease characteristics are summarized in Table 1. The median age at study entrance was 76years (range, 70–87). Twenty (29%) patients were aged >80 years and half of them were considered frail according to the IMWG frailty score.^14^ The presence of high-risk cytogenetic abnormalities was determined at time of study inclusion for 54 patients. Del(17p) and t(4;14) were found in 10 (18.5%) and 8 (10%) respectively. The median time from MM diagnosis and study enrolment was 28 months. Based on inclusion criteria, all patients received one prior line of therapy, and 52 (74%) patients had lenalidomide refractory disease and 26 (37%) had disease refractory to both lenalidomide and anti-CD38. Most common (>10%) front-line regimens were daratumumab lenalidomide dexamethasone (*n* = 26, 37%), bortezomib lenalidomide dexamethasone (*n* = 22, 31.5%) and lenalidomide dexamethasone (*n* = 13, 18.5%). At the data cut-off date for this analysis (April 14, 2024), treatment was ongoing in 31 (44%) patients and 39 (56%) patients discontinued therapy due to disease progression (*n* = 33, 47%), adverse event (AE) (*n* = 4, 6%) or death (*n* = 2, 3%).

### Efficacy

**FIGURE 1:**
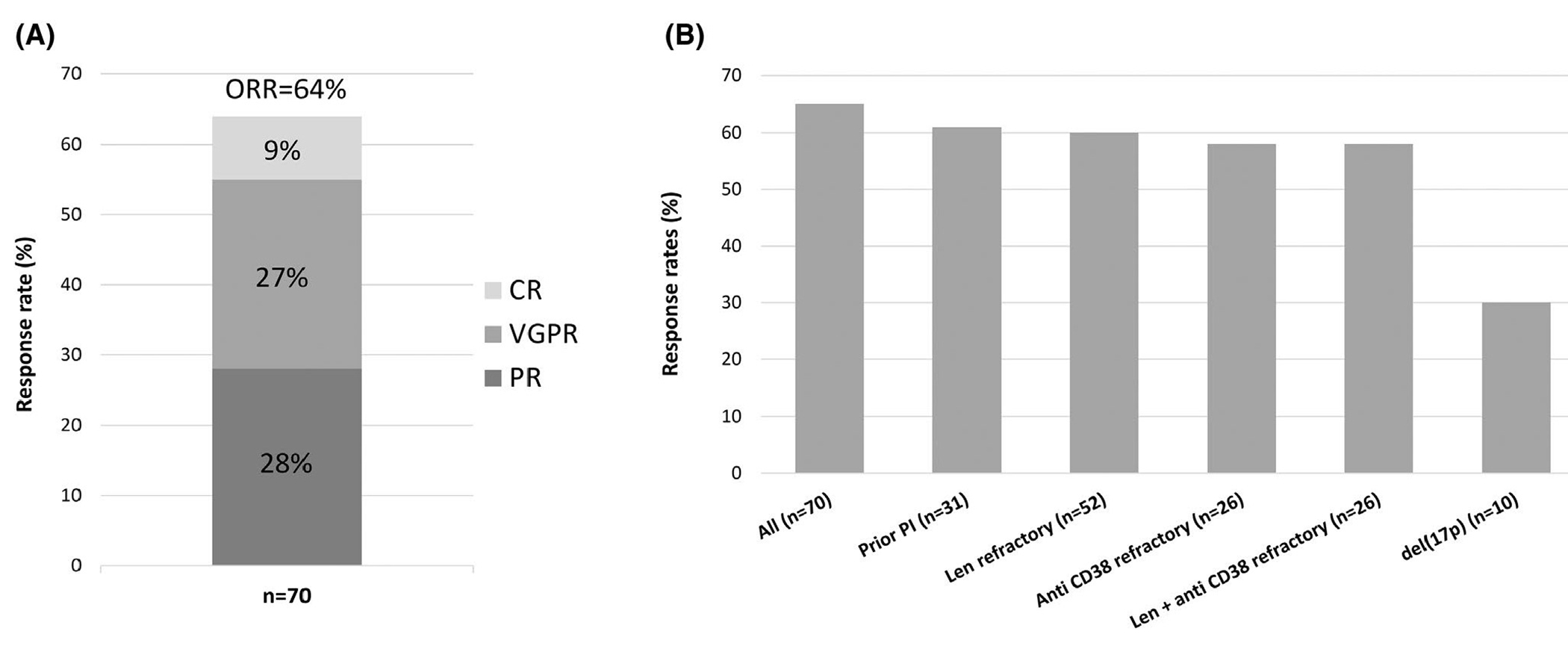
(A) Response rates (B) Subgroup analysis of response. CR, complete remission; Len, lenalidomide; ORR, overall response rate; PI, proteasome inhibitor; PR, partial response; VGPR, very good partial response.

**FIGURE 2:**
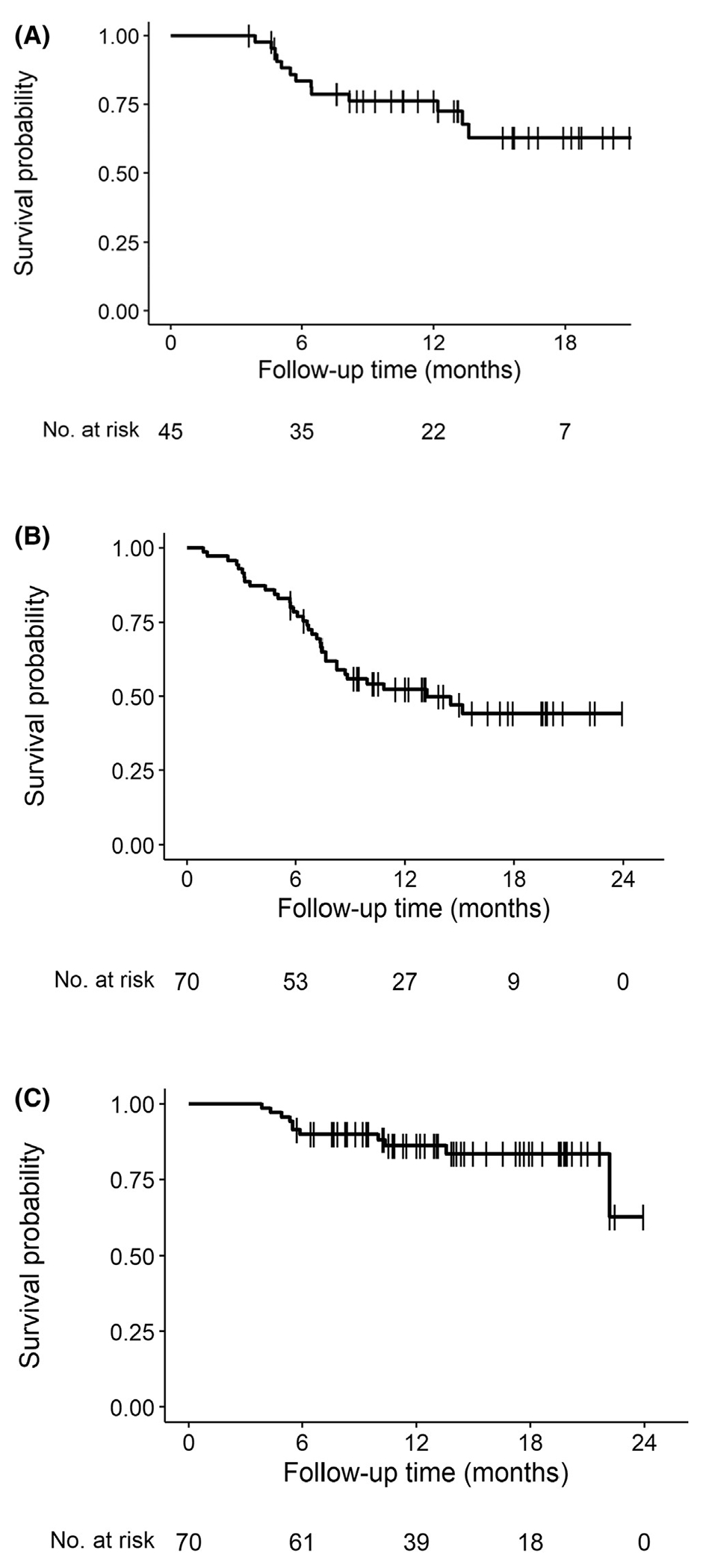
(A) Duration of response; (B) progression-free survival; (C) overall survival.

Response rates are summarized in Figure 1A. The overall response rate (ORR) was 64% (n = 45), including 36% (*n* = 25) with VGPR or better. Twenty patients achieved a partial response (PR), 19 achieved a VGPR and six patients achieved a CR. With a ≥VGPR rate of 36%, the study did not meet its primary objective (VGPR or better rate >45%). The ORR in patients refractory to lenalidomide (*n* = 52) and in patients refractory to lenalidomide and anti-CD38 (*n* = 26) was 60% and 58% respectively. Patients with del(17p) *n* = 10) had an ORR of 30%. Response rates in subgroups of patients are shown in Figure 1B. Among all responding patients, the median DOR was not reached (NR) and the 12-month DOR was 76% (95% CI 64%–90%) (Figure 2A). With a median follow-up of 14 months, the median PFS was 13 months (95% CI 8.2–NR) (Figure 2A). The median PFS was 9.9 months (95% CI 5.7–NR) in patients refractory to lenalidomide and anti-CD38 (*n* = 26). versus 15 months (95% CI 9.9–NR) in other patients (*n* = 44), *p* = 0.63 (Figure 2B). The median PFS was similar in frail patients (IMWG score ≥2) versus non-frail patients. The median OS was not reached and the 12-month OS was 86% (95% CI 78%–95%) (Figure 2C).

### Safety

At data cut-off, patients received a median of 10 cycles of I2D (range, 1–27). AEs occurring in at least 10% of patients (or at least 5% for grade 3–4 AEs) are described in Table 2. For haematological toxicity, neutropenia was the most frequent treatment-related AE, occurring in 34 (54%) patients, mainly grade 3 or 4. For non-haematological toxicity, the most common AEs were gastrointestinal (GI) disorders (*n* = 23, 36%), infection (*n* = 19, 30%) and fatigue (*n* = 14, 22%), mostly grade 1 or 2. Five patients presented with grade 3–4 infection (COVID-19 in two patients, pneumonia in two patients and septicaemia in one patient). Two patients died due to infection. Peripheral neuropathy was reported in 14 (22%), all grade 1 or 2. Six patients (9%) developed skin rash, including 3 with grade 3–4 (5%). One patient (1%) developed deep vein thrombosis. The incidence of grade >2 toxicities was similar in frail (IMWG score ≥2) and in non-frail patients. Overall, 4 (6%) patients discontinued treatment permanently due to treatment-related toxicity: skin rash (*n* = 1), cytopenia (*n* = 2) and peripheral neuropathy (*n* = 1). Dose reduction of iberdomide, ixazomib or dexamethasone occurred in 17 (24%), 18 (25%) and 7 (10%) patients respectively.

## DISCUSSION

The benefit of lenalidomide combined with anti-CD38 given until disease progression for the front-line therapy of transplant ineligible myeloma patients has been confirmed by three randomized trials.^2,15,16^ In the near future, most TI patients will present with MM refractory to lenalidomide and/or anti-CD38 at first relapse, and older, more frail patients represent a difficult-to-treat population. To the best of our knowledge, no prospective studies specifically addressed this population of patients. The present study included elderly (median 76years) patients at first relapse, with half of them being frail according to the IMWG frailty score.^14^ The majority (74%) of patients had lenalidomide refractory disease and 37% were refractory to both lenalidomide and anti-CD38. Most patients achieved a response (ORR = 64%) and the 12-month DOR was 76%. Overall response rates were similar in patients with lenalidomide + anti-CD38 refractory disease. While the study did not meet its primary objective (VGPR or better rate), the efficacy profile of I2D appears as favourable in this population of elderly patients with high-risk features. Indeed, the primary hypothesis of at least 45% VGPR rate was extrapolated from the PVd data at first relapse (OPTIMISSM trial). However, we did not expect at the time of study design to enrol a population with such high proportion of frail patients (50%), with a median time from diagnosis to enrolment of nearly 2 years and with lenalidomide (74%) and daratumumab (37%) refractory disease. With a median follow-up of 14months, the median PFS was above 1 year. Interestingly, a similar PFS was observed in patients with lenalidomide and anti-CD38 refractory disease. Regarding safety, I2D appeared to be a convenient and well-tolerated oral combination, with a low rate of grade 3–4 non-haematological AEs. The most common grade 3–4 toxicity was neutropenia, as previously reported with iberdomide.^7^ Common (>20%) non-haematological AEs (GI disorders, fatigue, infection and peripheral neuropathy) were mostly grade 1 or 2. No patients developed grade 3–4 peripheral neuropathy, that compared favourably with PVd combination (>8%).^4^ The use of low-dose and short-duration dexamethasone in this study might have contributed to the low rate of grade 3–4 steroid-related toxicities (i.e. infection, hypertension, psychiatric disorders) in this population. To the best of our knowledge, no safety/efficacy data with other therapeutic strategies are available in this population of elderly/frail MM patients at first relapse, notably with a high proportion being refractory to lenalidomide + anti-CD38. The ALLIANCE A061212 trial evaluated ixazomib, pomalidomide and dexamethasone in 38 MM patients at first relapse.^9^ The ORR and median PFS were 63.2% and 20.3 months respectively. However, patients in the latter study were younger (median age 66years) with no patient exposed/refractory to daratumumab. The triplet pomalidomide ixazomib dexamethasone also demonstrated favourable efficacy safety profile in high-risk RRMM patients.^10^ Several regimens are suitable for patients with lenalidomide/anti-CD38 refractory disease (summarized in Table 3). In a recent phase 3 trial including patients refractory to lenalidomide/antiCD38, the median PFS with PVd was 12.7 months.^17^ Moreover, PVd is associated with a significant incidence of bortezomib-related peripheral neuropathy (48%, including 8% grade 3–4 in the OPTIMISMM trial)^4^ In the CANDOR and IKEMA trials, the median PFS in the Kd arm in lenalidomide-refractory patients was 11 and 15months respectively.^18,19^ Moreover, bi-weekly carfilzomib could be challenging in an elderly frail population. More recently, belantamab mafodotin (Bela) in combination with Pd or Vd demonstrated favourable efficacy in RRMM patients, but was associated with a high incidence of clinically significant ocular AEs; challenging in elderly/frail patients.^17,20^ Cilta-cel and ide-cel demonstrated strong activity in RRMM from first or second relapse.^21,22^ However, a very scarce proportion of patients were aged over 70years and the safety of chimeric antigen receptor T-cell therapy targeting B-cell maturation antigen (BCMA) in older patients still needs to be demonstrated. In triple-class exposed advanced RRMM patients, BCMA and GPRC5D bispecific antibodies also demonstrated high response rates and PFS.^23 25^ Similarly, safety of these agents (i.e. with respect to risk of cytokine release syndrome, infections, skin toxicity, dysgeusia) still needs to be determined in elderly patients.

In conclusion, iberdomide ixazomib and short duration of dexamethasone demonstrated a favourable efficacy/safety profile in elderly patients at first relapse, including in those with lenalidomide/anti-CD38 refractory disease. Randomized phase 3 studies should be dedicated to elderly patients with lenalidomide/anti-CD38 refractory disease to determine the optimal benefit/risk strategy in this difficult-to-treat population.

## Figures and Tables

**TABLE 1:** Patient demographic and baseline disease characteristics.

Characteristic	Study population *n* = 70
Sex: Male/female, *n*	38/32
Median age (range), years	76 (70–87)
Age >80, *n* (%)	20 (29)
ECOG PS, *n* (%)	
0–1	66 (94)
2	4(6)
IMWG frailty score, *n* (%)	
0–1 (fit, intermediate)	35 (50)
≥2 (frail)	35 (50)
High-risk cytogenetic abnormalities,^a^ *n* (%)	
Del(17p), *n* (%)	10 (18.5)
t(4;14), *n* (%)	8(15)
Median time from diagnosis to study enrolment, months (range)	28 (5–130)
Prior therapy in first line, *n* (%)	
Proteasome inhibitor	31 (44)
Lenalidomide	61 (87)
Anti-CD38	26 (37)
Refractory status, *n* (%)	
Lenalidomide	52 (74)
Anti-CD38	26 (37)
Lenalidomide and anti-CD38	26 (37)

Abbreviations: ECOG PS, Eastern Cooperative Oncology Group performance status; IMWG, International Myeloma Working Group; ISS, International Staging System; R-ISS, revised ISS.

a Assessed in 54 patients.

**TABLE 2:** Adverse events (≥10%) reported through induction and consolidation.

Adverse event	Any grade patients, *n* (%)	Grade 3/4 patients, *n* (%)
Haematological		
Anaemia	7(11)	1 (2)
Neutropenia	34 (54)	29 (46)
Thrombocy topen ia	7(11)	6(9)
Non-hematologic		
GI disorder	23 (36)	3(5)
Infection	19 (30)	5(8)
Fatigue	14 (22)	2(4)
Insomnia/sleep disorders	14 (22)	0
Peripheral neuropathy	14 (22)	0
Muscle spasms	21 (42)	2(4)
Skin rash	6(9)	3(5)

Abbreviation: GI, gastrointestinal.

**TABLE 3:** Summary of key options for patients with 1 to 3 prior lines of therapy and MM refractory to lenalidomide and antiCD38.

Clinical trial	Population	Treatment	Median age	Proportion of len refractory (%)	Proportion of anti-CD38 exposed (%)	Median PFS (months)
Optimismm^4,26^	1–3 PL/frail^a^	PVd	74	67.7	-	9.7
DREAMM-8^17^	1–3 PL	PVd	68	76	29	12.7
IKEMA^18^	1–3 PL	Kd	63	32	1	19.1
CANDOR^19^	1–3 PL	Kd	64	36	-	15.2
DREAMM-8^17^	1–3 PL	BelaPd	67	81	25	71% at 12 months
DREAMM-7^20^	1–3 PL	BelaVd	65	33	1	36.6
KARMMA-3^22^	2–4 PL	Ide-cel	63	73	95	13.3
CARTITUDE-4^21^	1–3 PL	Cilta-Cel	61.5	100	25.5	75.9% at 12 months

Abbreviations: Bela-Pd, belantamab-mafodotin pomalidomide dexamethasone; Bela-Vd, belantamab-mafodotin bortezomib dexamethasone; Kd, carfilzomib dexamethasone; PFS, progression-free survival; PL, prior line; PVd, pomalidomide bortezomib dexamethasone.

a Subgroup analysis in frail patients.

## Data Availability

Individual participant data will not be shared.
